# Biochemical evidence of both copper chelation and oxygenase activity at the histidine brace

**DOI:** 10.1038/s41598-020-73266-y

**Published:** 2020-10-01

**Authors:** Søren Brander, Istvan Horvath, Johan Ø. Ipsen, Ausra Peciulyte, Lisbeth Olsson, Cristina Hernández-Rollán, Morten H. H. Nørholm, Susanne Mossin, Leila Lo Leggio, Corinna Probst, Dennis J. Thiele, Katja S. Johansen

**Affiliations:** 1 Department of Geoscience and Natural Resource Management, University of Copenhagen, 1958 Frederiksberg, Denmark; 2 Division of Chemical Biology, Department of Biology and Biological Engineering, Chalmers University of Technology, Kemivägen 10, 412 96 Gothenburg, Sweden; 3 Department of Plant and Environmental Sciences, University of Copenhagen, 1871 Frederiksberg, Denmark; 4 Division of Industrial Biotechnology, Department of Biology and Biological Engineering, Chalmers University of Technology, Kemivägen 10, 412 96 Gothenburg, Sweden; 5 Novo Nordisk Foundation Center for Biosustainability, Technical University of Denmark, 2800 Kgs. Lyngby, Denmark; 6 Centre for Catalysis and Sustainable Chemistry, Department of Chemistry, Technical University of Denmark, 2800 Kgs. Lyngby, Denmark; 7 Department of Chemistry, University of Copenhagen, 2100 Copenhagen Ø, Denmark; 8 Department of Biochemistry, Pharmacology and Cancer Biology and Molecular Genetics and Microbiology, Duke University School of Medicine, Durham, NC 27710 USA

**Keywords:** Biochemistry, Biological techniques, Biotechnology, Chemical biology, Structural biology

## Abstract

Lytic polysaccharide monooxygenase (LPMO) and copper binding protein CopC share a similar mononuclear copper site. This site is defined by an N-terminal histidine and a second internal histidine side chain in a configuration called the histidine brace. To understand better the determinants of reactivity, the biochemical and structural properties of a well-described cellulose-specific LPMO from *Thermoascus aurantiacus* (TaAA9A) is compared with that of CopC from *Pseudomonas fluorescens* (PfCopC) and with the LPMO-like protein Bim1 from *Cryptococcus neoformans.* PfCopC is not reduced by ascorbate but is a very strong Cu(II) chelator due to residues that interacts with the N-terminus. This first biochemical characterization of Bim1 shows that it is not redox active, but very sensitive to H_2_O_2_, which accelerates the release of Cu ions from the protein. TaAA9A oxidizes ascorbate at a rate similar to free copper but through a mechanism that produce fewer reactive oxygen species. These three biologically relevant examples emphasize the diversity in how the proteinaceous environment control reactivity of Cu with O_2_.

## Introduction

Mechanisms for harnessing copper in living tissues have evolved throughout biology. Excess copper leads to production of reactive oxygen species and inactivate vital enzymes by displacement of iron atoms and is therefore toxic to living cells^1,2^. Copper is often found coordinated by amino acid sidechains of proteins that function in maintaining copper homeostasis as Cu-chelators^3^. However, copper is also an essential co-factor in important classes of enzymes such as laccase, Cu–Zn superoxide dismutase, cytochrome c oxidase, particulate methane monooxygenase (pMMO) and lytic polysaccharide monooxygenase (LPMO)^3,4^. Intriguingly, the Cu-coordination sphere of Cu-chelators and Cu-enzymes appear in some cases to involve the same amino acid residues. In our experience^5,6^, it is very difficult to biochemically or structurally determine if a novel Cu-protein is an enzyme or a Cu-chelator. The ongoing scientific controversy regarding the active site of pMMO^7–9^ is reflecting the same problem. Absence of activity in metallo-enzyme assays can in principle be due to a number of factors including lack of suitable reducing agent or substrate. However, it can also be caused by the inability of the metal-cofactor to undergo redox-cycling.

**Figure 1:**
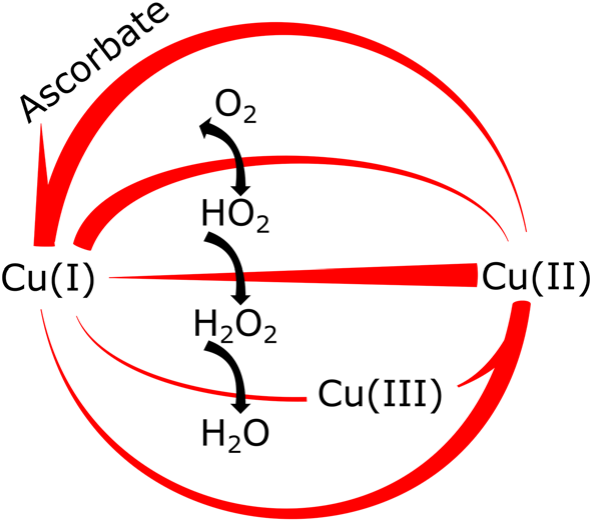
Schematic illustration of copper-catalysed oxidation of ascorbate coupled to the reduction of oxygen to water based on Pham et al. 2013^10^. The oxygen species are written in their neutral form. The thickness of the connecting lines between copper species indicates the individual rates of that particular conversion. The figure was drawn in Inkscape 0.92.4.

The complex chemistry of aerobic oxidation of ascorbate by labile copper (termed free copper in this work) has been well studied. A simplified schematic illustration of copper-catalysed oxidation of ascorbate coupled to the reduction of O_2_ to water based on Pham et al. 2013^10^ is shown in Fig. 1. At circumneutral pH Cu(II) is readily reduced by ascorbate but the following reduction of molecular oxygen to superoxide is much disfavoured thermodynamically and produce superoxide in trace amounts only. Superoxide is reduced by a fast and efficient reaction with Cu(I) to form H_2_O_2_ and it is this fast reduction of superoxide that drives reduction of molecular oxygen by virtue of Le Chatelier’s principle. Oxidation of Cu(I) by H_2_O_2_ produce water but also the highly reactive chemical species Cu(III). The chemistry is complicated by reaction of the intermediates with each other and with ascorbate but the outcome is rapid oxidation of ascorbate. Even trace impurities of copper lead to abiotic oxidation of ascorbate^11,12^. In this study, the fundamental knowledge about inorganic Cu-chemistry is used as the platform for the design and interpretation of the reactivity of Cu-proteins.

A special case of Cu-coordination, the copper–histidine brace^4,13^, is found in the active site of LPMOs. They are a class of enzymes that cleave glycoside bonds in the major forms of polysaccharides including cellulose, starch, hemicellulose and chitin^14–16^. This site contain a mononuclear copper coordinated by an N-terminal histidine side chain, the N-terminus and a second histidine side chain^4^. This configuration leaves an open coordination position for the co-substrate O_2_ and enables one-electron reduction of O_2_ by concomitant oxidation of enzyme-bound Cu(I)^17^. This allows for further reduction of the enzyme–substrate complex followed by cleavage of the glycosidic bond. Catalysis is fine-tuned by cooperative binding of the polymeric substrate and O_2_^18^. This allows for further reduction of the enzyme–substrate complex followed by cleavage of the glycosidic bond. However, activated oxygen in the form of hydrogen peroxide has also been shown to act as co-substrate^19–21^.

**Figure 2:**
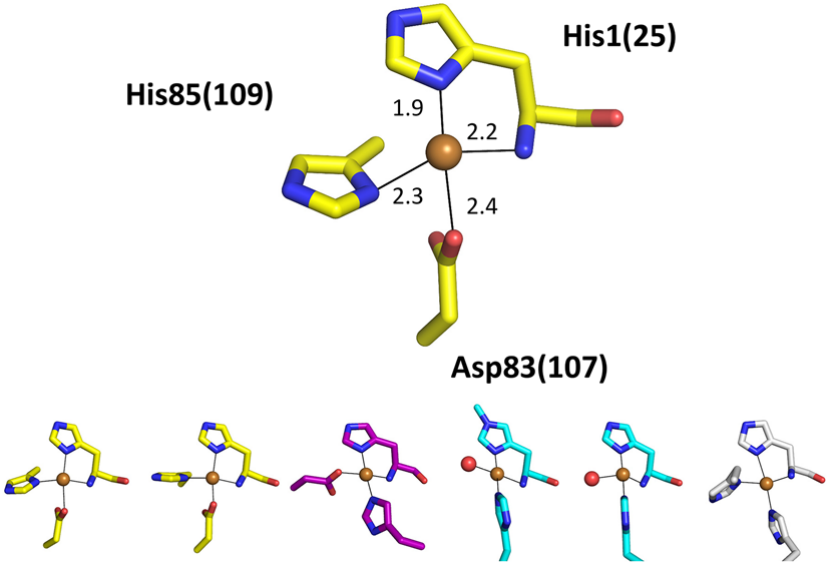
Structures of copper–histidine braces in different proteins. CopC proteins shown are in yellow, X325 in purple, LPMOs in light blue, and pMMO in white. The axial copper ligands are not shown to improve clarity. The structures are shown in the same orientation after alignment of the N-terminal histidines. Top: PfCopC from (PDB code 6NFQ) Distances between the copper atom and the proteins are in Å.) Bottom from left to right: MtCopC from Methylosinus trichosporium OB3b (PDB code 5ICU); TpCopC from Thioalkalivibrio paradoxus ARh 1 (PDB code 5N1T); LaX325 from Laetisaria arvalis (PDB code 6IBJ); TaAA9A (PDB code 2YET); EfAA10A from Enterococcus faecalis (PDB code 4ALC); and Cu site B in pMMO from Methylomicrobium alcaliphilum 20Z (PDB code 6CXH). Drawn in PyMol 2.3.1.

As illustrated in Fig. 2 copper–histidine brace can be identified in several other proteins of which LPMOs are the only ones to have confirmed activity at this site. To understand better the determinants of reactivity of LPMO and other copper–histidine brace proteins, we report a structural and biochemical comparison of three mononuclear Cu-proteins. These are the bacterial Cu chaperone CopC from *Pseudomonas fluorescens* (PfCopC), the LPMO-like protein Bim1 involved in brain colonization by *Cryptococcus neoformans*, and the well characterised cellulose specific AA9 LPMO from *Thermoascus aurantiacus* (formerly TaGH61, now TaAA9A)^4,22^. This is the first biochemical characterisation of Bim1. These molecules constitute a series of Cu-coordination variants of the copper–histidine brace that shed light on the fundamental differences in chemistry between them. In particular, we have investigated how the three Cu-proteins respond to the biologically relevant reductant ascorbate.

## Results

### Preparation, copper binding and structural analysis of Cu-CopC

**Figure 3:**
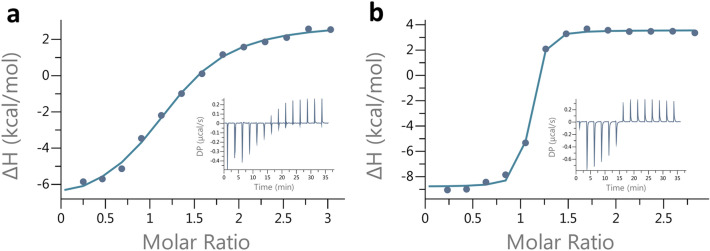
ITC titrations of 25 µM apo-proteins with [CuI(MeCN4)]^+^ in 40 mM MES, 150 mM NaCl pH 6.6 at 25 °C. Under these conditions (**a**) PfCopC shows a weak but specific binding that suggests K_d_ = 1.1 × 10^–5^ M and (**b**) TaAA9A shows a stronger binding and a K_d_ ≤ 3.4 × 10^–7^ M.

The coding DNA sequence of PfCopC including the native signal peptide (GenBank access no. WP_015884740.1) was expressed in *E.coli* and chromatographically purified. Apo-PfCopC has an extremely high affinity for Cu(II) but does not compete with the ligand bicinchoninic acid (BCA) for Cu(I) under anaerobic conditions^23^. This was confirmed by anaerobic Isothermal Titration Calorimetry (ITC) of Cu(I)-(BCA)_2_ in apo-PfCopC (SI Fig. 1a). However, titration of the weaker [CuI(MeCN4)]^+^ complex in apo-PfCopC resulted in thermograms consistent with formation of Cu(I)-PfCopC with a suggested K_d_ = 1.1 × 10^–5^ M (Fig. 3a, SI Fig. 1b,c). The native crystal structure of PfCopC (Fig. 1) shows clearly that His1, His85 and Asp83 bind Cu(II)^24^. Furthermore, Glu27, which is highly conserved in CopC proteins (SI Fig. 2), is within hydrogen bonding distance from the N-terminus. Using this crystal structure of PfCopC for a Dali search^25^ identifies the closest structural neighbour with a correctly processed N-terminus and a metal at the copper binding site as PsCopc (PDB code 2C9Q, Z-score of 16.0, 47% sequence identity and 1.3 Å rmsd over 94 aligned Cα atoms). In PsCopC (2C9Q) the coordination to the Cu is slightly different in that the Asp-Cu interaction is water mediated^26^.The next relevant structural neighbour is MtCopC from *Methylosinus trichosporium* OB3b^27^, PDB code 5ICU, Z-score of 15.7, 32% sequence identity and 1.4 Å rmsd over 94 aligned residues, with a very similar Cu(II) binding site (Fig. 1). This is closely followed by chain W in PDB code 5N1T, representing the structure of Cu-bound TpCopC from *T. paradoxus*^28^ (complexed with a sulphide dehydrogenase), with a Z score of 14.4, 34% sequence identity and 1.8 Å rmsd over 92 aligned residues, also with a very similar Cu-binding site.

### Preparation and structural analysis of Cu-Bim1

Bim1 was expressed in *P. pastoris* and chromatographically purified as described previously^5^. The C-terminal His tag was removed from the protein using TEV protease. The structure of LaX325 has recently been determined by X-ray crystallography^6^ and the structure of Bim1 (36% identical in sequence) was modelled on this basis (Fig. 2)^5^. The homology model of Bim1 supports similar coordination by a His-brace (including N-terminus) and an Asp residue on a Gly-rich and thus potentially flexible loop. Mutagenesis studies have confirmed the functional importance of these residues in copper import. In this respect, the Cu binding site resembles the CopC binding site, but with the positions of Asp and the internal His swapped (Fig. 2). The copper binding site of LaX325, and thus presumably also Bim1, resembles closely the geometry of the Cu(II) site of LPMOs including TaAA9A. An important difference in LPMOs is that the Asp ligand site is instead free for access by an exogenous ligand, and often axial coordination is provided by a Tyr. The geometrical similarity includes the observed bond lengths and angles θ1, θ2 and θ3 as defined by Vu and Ngo 2018^29^. The θ1 angle (NH_2_-Cu-His1Nδ) lies also between 88.5° and 92.1° in the PfCopC structure, thus also within the values observed for Cu(II)-LPMOs, while the two other angles are not directly comparable because of the swapping of position of Asp and His ligand.

### Preparation of Cu-TaAA9A and copper binding analysis

TaAA9A was heterologously expressed as described previously^4^. The sample, kindly provided by Novozymes A/S, was purified using ion-exchange chromatography. An ITC assay was carried out to determine the binding capacity of CuSO_4_ for this batch of enzyme. It was found that a relative amount of 0.75 sub-stoichiometric CuSO_4_ gave a fully loaded preparation of Cu(II)-TaAA9A without any free copper in excess (SI Fig. 3a, b). ITC titration of [CuI(MeCN4)]^+^into apo-TaAA9A under anaerobic condition was consistent with formation of Cu(I)-TaAA9A with a K_d_ ≤ 3.4 × 10^–7^ M.(Fig. 3b).

### Neither PfCopC nor Bim1 degrade phosphoric-acid-swollen cellulose

**Figure 4:**
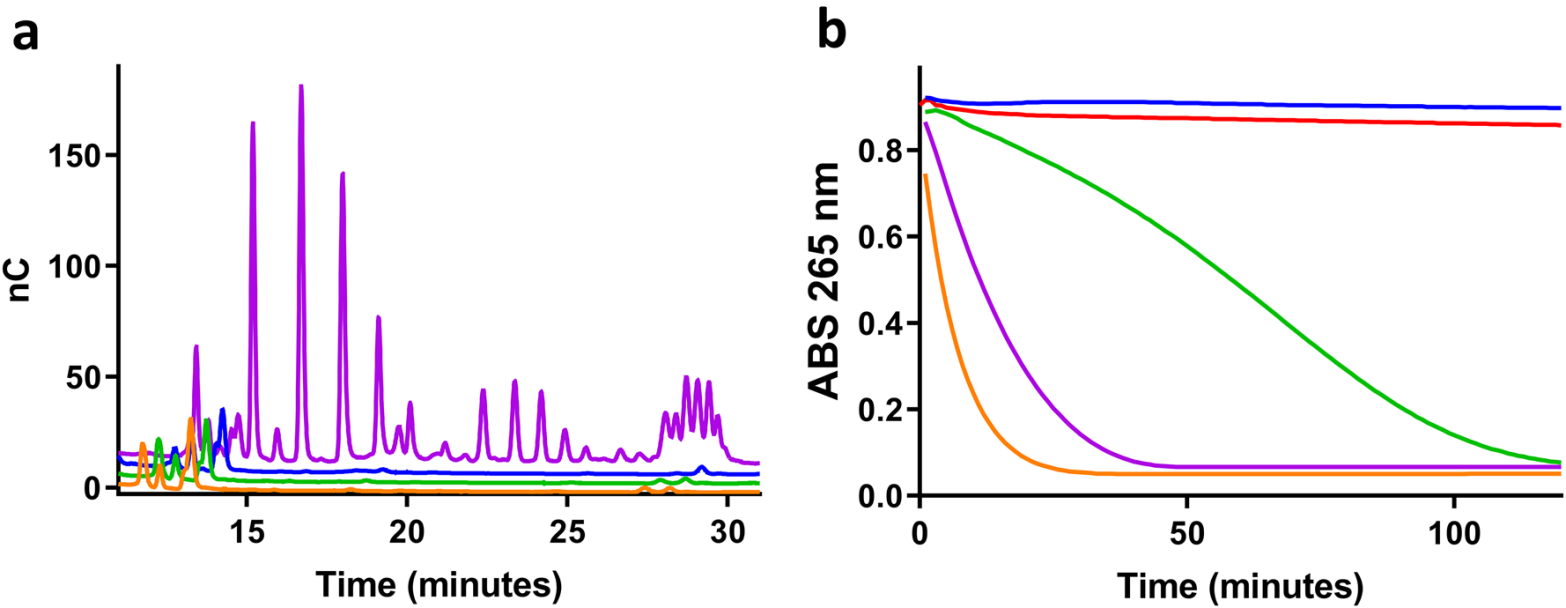
Comparison of LPMO-like activities in MES buffer pH 6.6. 1 µM PfCopC (blue), Bim1 (green), TaAA9A (purple) were preloaded overnight with 0.5 µM CuCl_2_ and compared with non-enzymatic samples of 0.5 µM CuCl_2_ (orange) or buffer (red). (**a**) HPAEC profile of oligo-saccharides produced after 24 h incubation of enzymes with PASC and 1 mM ascorbic acid. (**b**) Progress curves showing the disappearance of the specific 265 nm band during oxidation of 100 µM ascorbate.

Enzymatic assays showing the activity of Cu-TaAA9A on cellulose are well established. We evaluated the activity of Cu-PfCopC , Cu-Bim1 and Cu-TaAA9A in parallel using phosphoric-acid-swollen cellulose (PASC) as substrate and ascorbate as reductant. Chromatograms of the released cello-oligosaccharides after incubation at 40 °C are shown in Fig. 4a. The product profile in the Cu-TaAA9A-treated sample is as expected, showing a combination of oxidized oligomers (peaks with retention times longer than 20 min) and oligomers that are identical to hydrolysis products (retention times of 12–20 min). Samples treated with Cu-Bim1 or Cu-PfCopC contained no soluble cello-oligosaccharides.

### Ascorbate assay

**Figure 5:**
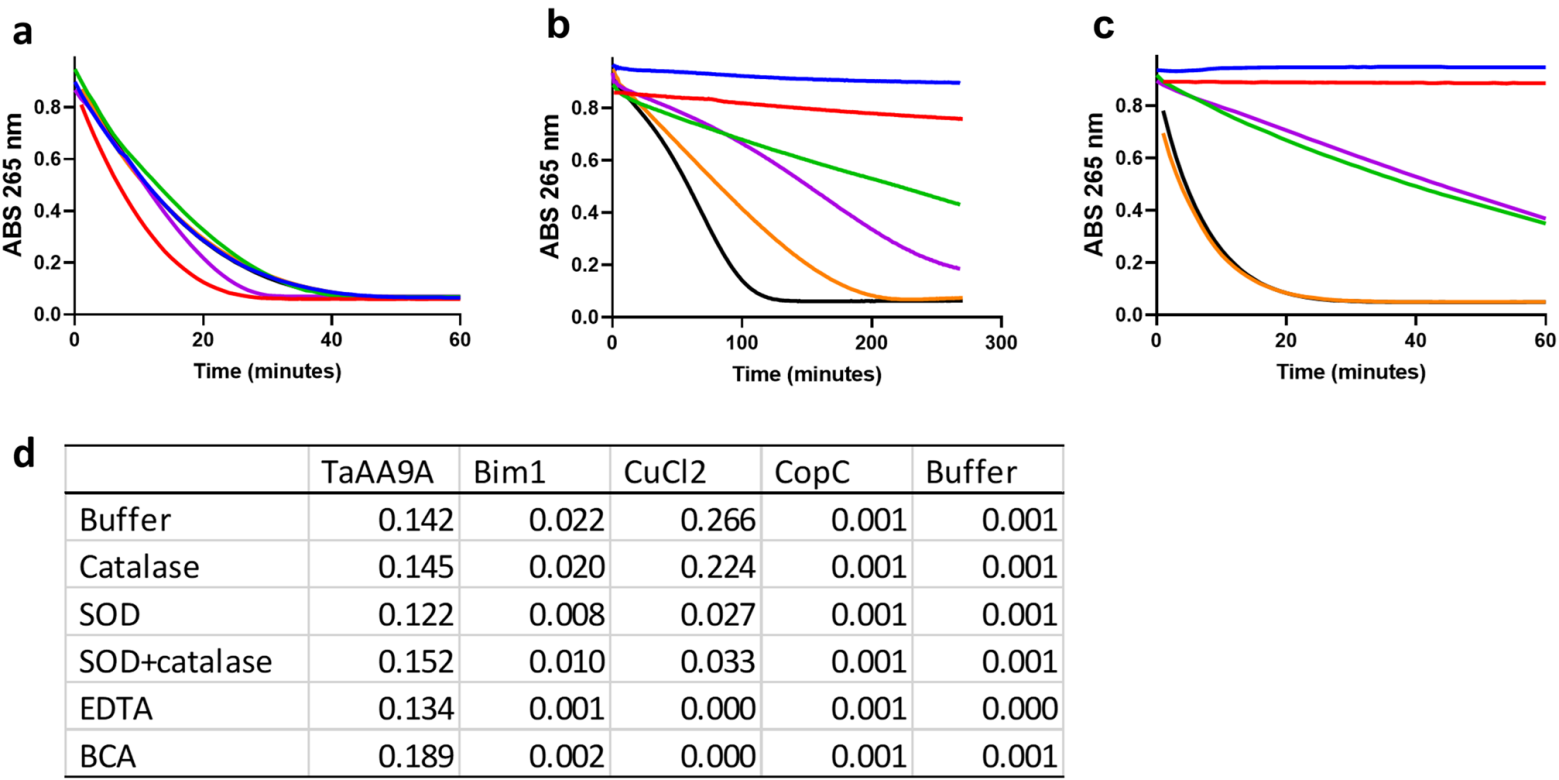
Oxidation of ascorbic acid in combination with different scavengers of oxygen species. (**a**) 1 µM TaAA9A, (**b**) 1 µM Bim1, and (**c**) buffer was equilibrated with 0.5 µM CuCl_2_ overnight and tested together with BCA (red), EDTA (blue), 10 nM superoxide dismutase and 1 nM catalase (green), 10 nM superoxide dismutase (purple), 1 nM catalase (orange) and without scavengers (black). 20 mM MES buffer pH 6.6 and 100 µM ascorbic acid, incubated at 26 °C. The absorption was read every minute with intermittent shaking. (**d**) Initial turnover frequency (s^-1^) of ascorbate oxidation determined by an approximate linear fit to the progress curve. The values for CopC and negative controls with buffer only are included in the table.

A fundamental requirement for Cu-enzymes is that the protein-bound Cu undergoes redox cycling during catalysis. This basic redox property of the three Cu-proteins was probed and compared using ascorbate as reductant. The oxidation of ascorbic acid into either the ascorbate radical or dehydro-ascorbate results in complete loss of absorbance at 265 nm ^30^. Such an assay has been used to investigate the redox chemistry of the Aβ16 peptide related to Alzheimer’s disease^31^. Many LPMOs, including TaAA9A, are known to be reduced by ascorbate and to release reactive oxygen species when reacted with ascorbate under aerobic conditions^22,32^. We used this assay to compare the ascorbate oxidizing activity of PfCopC, TaAA9A, and Bim1 in MES buffer at pH 6.6. (Fig. 4b). No ascorbate oxidation is seen with Cu-PfCopC. The apparent ascorbate oxidation turnover frequency by Cu-TaAA9A (0.14 s^-1^) appears at first to be lower than, but still in a similar range to, the oxidation of ascorbate catalysed by free copper (0.27 s^-1^, Fig. 5a,d). In contrast, the sigmoidal progress curve for Bim1 shows a much slower and somewhat delayed oxidation of ascorbate. The sigmoidal progress curve is indicative of the involvement of a rate-accelerating step in the reaction.

The Cu redox chemistry involves several copper and oxygen species that react with widely different rates^10^. Known reactive oxygen species (ROS) scavengers were therefore used to probe how the proteinaceous environment control different parts of the Cu redox cycle in the three proteins. The effects of mannitol as a scavenger of OH^•^, superoxide dismutase (SOD) which removes HO_2_^•^ while producing H_2_O_2_, and catalase, which terminates ROS cascades by turning H_2_O_2_ into water and O_2_ were tested (Fig. 5). Mannitol had no effect on the ascorbate oxidation progress curves under any of the conditions tested (data not shown) in agreement with previously reported data for un-ligated copper^10^. SOD reduced the turnover frequency of ascorbate oxidation by free Cu, but had very limited effect on the reaction catalysed by Cu-TaAA9A. In contrast, SOD had a strong retarding effect on ascorbate oxidation by Cu-Bim1. Catalase had no effect on ascorbate oxidation in the samples with free Cu or TaAA9A. Again, the Cu-Bim1 samples with catalase stand out, showing a clear retarding effect on the progress curve, which became almost linear (Fig. 5b). The combination of SOD and catalase with Cu-Bim1 is additive. These results are in agreement with a mechanism in which copper is progressively released from Cu-Bim1 but retained by Cu-TaAA9A.

This observation of copper release was substantiated by the effect of the Cu(II) chelator EDTA and the Cu(I) chelator BCA on the oxidation of ascorbate during incubation. Both chelators completely inhibited the oxidation of ascorbate by both CuCl_2_ and Cu-Bim1, while having a relatively small effect on the reaction catalysed by Cu-TaAA9A. In fact, BCA led to a small increase in the rate of ascorbate oxidation by Cu-TaAA9A. It should be noted that this effect can be explained by the elimination of a fast (3 × 10^9^ s^-1^)^33^ reaction between un-ligated Cu(I) and Cu(III)-TaAA9A (Fig. 5a). This reaction would compete with electron transfer from ascorbate to Cu(III)-TaAA9A to generate Cu(II)-TaAA9A. The estimated copper dissociation constant for Cu(II)-TaAA9A is < 1 nM^4^ but ≤ 0.3 µM for Cu(I)-TaAA9A (Fig. 3b) and must be taken into account as the source of free Cu(I) despite the fact that it is low^4^.

### Production of H_2_O_2_

**Figure 6:**
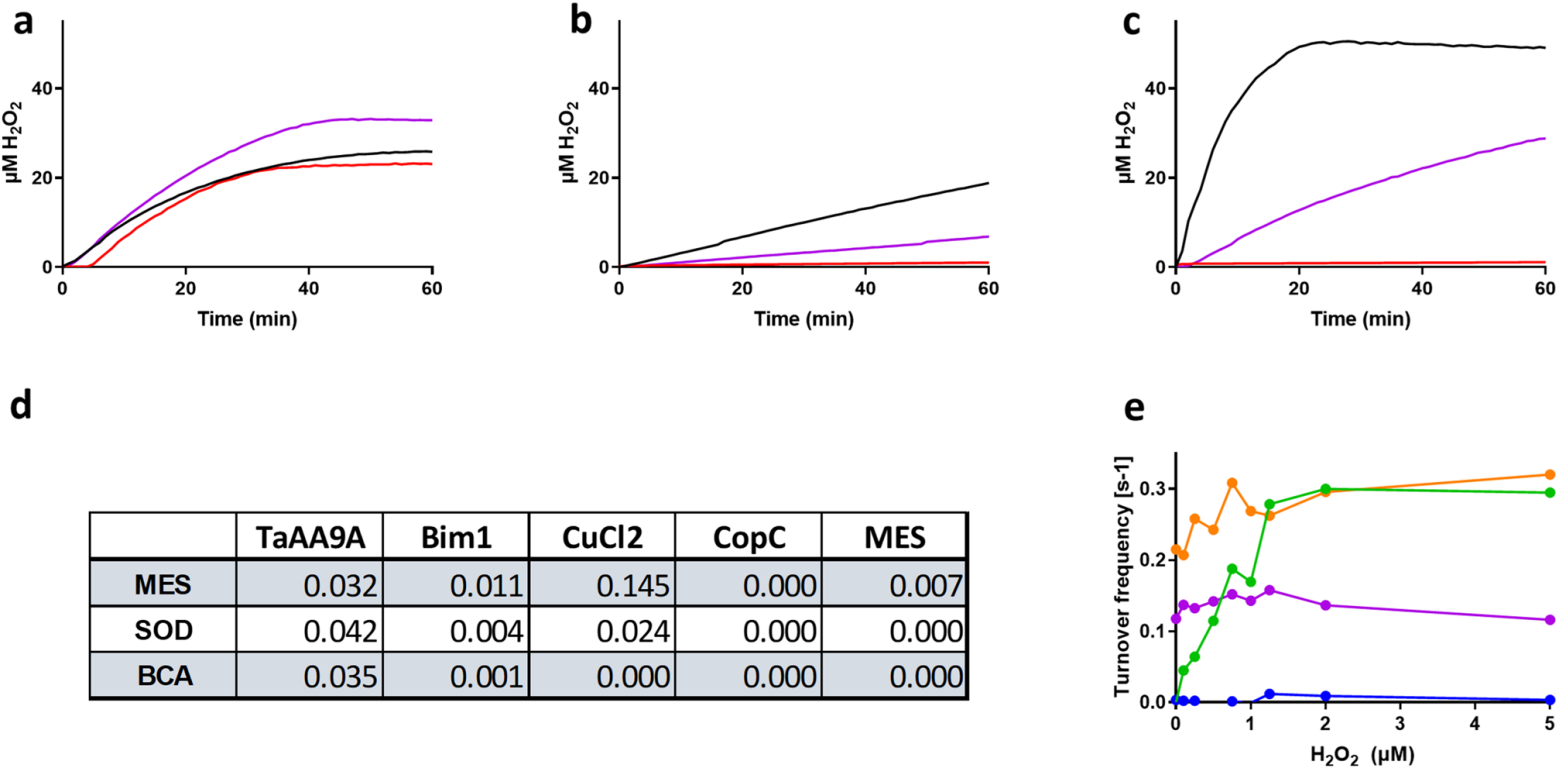
Production of H_2_O_2_ from 100 µM ascorbic acid in samples with 0.5 µM CuCl_2_ loaded (**a**) 1 µM TaAA9A, (**b**) 1 µM Bim1 or (**c**) buffer tested in presence of 20 µM bicinchoninic acid (red), 10 nM superoxide dismutase (purple) and without scavengers (black). (**d**) Table of turnover frequency of H_2_O_2_ production given in (s^-1^). (**e**) Initial turnover frequencies (s^-1^) of ascorbate oxidation by 0.5 µM CuCl_2_ loaded PfCopC (blue), Bim1 (green), TaAA9A (purple) or buffer (orange) after preincubation with 0–5 µM H_2_O_2_ for 30 min.

In order to explain the effect of catalase on the ascorbate turnover frequency (Fig. 5) we determined the availability of its substrate H_2_O_2_. The samples were prepared similarly to the ascorbic acid assay described above, but also contained 0.1 U/mL horseradish peroxidase and 50 µM Amplex UltraRed reagent. As shown in Fig. 6a-d, no H_2_O_2_ production was detected when BCA was combined with CuCl_2_, Cu-PfCopC or Cu-Bim1. Un-ligated copper produced H_2_O_2_ at high rates. Cu-TaAA9A produced H_2_O_2_ at a considerably lower, but still significant, rate. (SI Fig. 4 and discussed below). Cu-Bim1 produced H_2_O_2_ at a low rate, which was extinguished by BCA. It thus appears that the H_2_O_2_ production detected from this protein is primarily due to labile Cu that has leaked from the coordination site. Furthermore, the observed effect of catalase on ascorbate oxidation by Bim1 (Fig. 5) suggests that the H_2_O_2_ produced is involved in accelerating the release of Cu from the protein.

### Bim1 is highly sensitive to H_2_O_2_ in the ascorbate oxidation assay

The apparent sensitivity of Cu-Bim1 to H_2_O_2_ in the ascorbate oxidation assay was further evaluated. The turnover frequency of ascorbate oxidation was drastically increased in a H_2_O_2_ concentration-dependent manner. The oxidation rate resembles that of 0.5 µM free Cu as the concentration of H_2_O_2_ approached the concentration of Cu-Bim1 (Fig. 6e). These findings confirm that Cu is released into the solution from Cu-Bim1 under the conditions tested. This suggests that the histidine brace of Bim1, which coordinates to the Cu(II), is compromised. PfCopC and TaAA9A are not sensitive to H_2_O_2_ under these conditions.

### PfCopC is not reduced by ascorbate

Intrigued by the apparent lack of redox activity, the basic redox property of Cu-PfCopC was studied. Cu(II) shows a d-d charge-transfer band at 600 nm that we used to directly measure the redox state. As expected from the ascorbate assays, 0.5 mM of PfCopC was not reduced by 1 mM ascorbate in aerobic or anaerobic conditions (SI Fig. 5a) and this is consistent with the ascorbate turnover frequency being limited by Cu(II) reduction rather than re-oxidation in Cu-PfCopC. However, the addition of 2.5 mM DTT did reduce PfCopC (SI Fig. 5b). PfCopC contains one tryptophan and the intrinsic fluorescence of Trp 77 is quenched by the nearby Cu(II) site. This indirect measure confirmed that DTT but not ascorbate reduce PfCopC. The fluorescence peak position did not change which shows that the protein was folded during both treatments (SI Fig. 5c).

### Site-directed amino acid variants of PfCopC

Three amino acid variants of PfCopC were prepared to study their effect on the Cu-chelating property. Each of the amino acid residues Ala2, His3 and Glu27 has in previous studies been proposed to be involved in the extremely low K_d_ of PfCopC for Cu(II)^23,24,26,28^ (see discussion). All three residues are highly conserved among PfCopC homologues (SI Fig. 2). We were particularly interested in the function of E27 in PfCopC that interacts with the N-terminus^26,28^. This Glu residue is highly conserved among most CopCs, but not as much in the large but less well-characterised sub-family of CopC represented by MtCopC^27^. Amino acid sequence conservation of homologues of PfCopC and MtCopC were mapped onto the respective structures to cast further light on the role of the Glu residue (SI Fig. 6).

**Figure 7:**
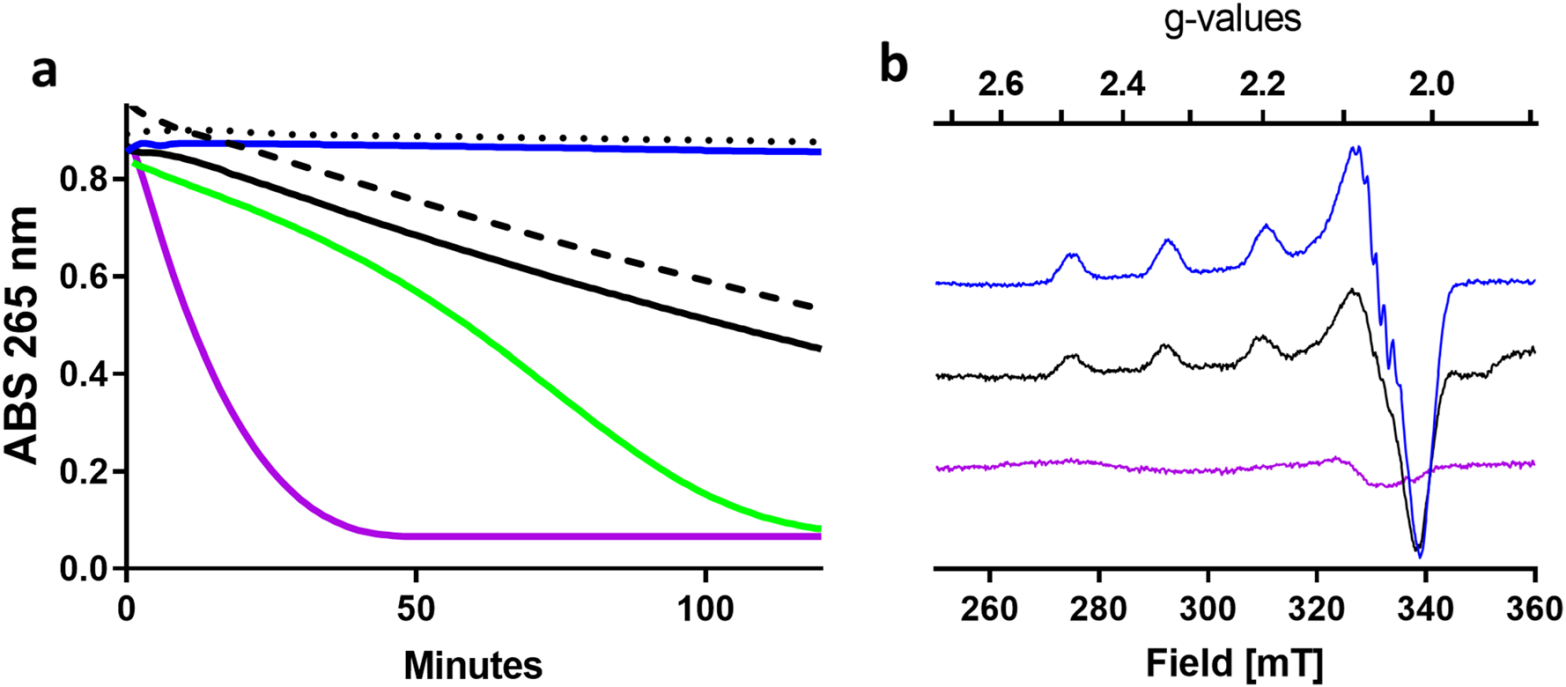
UV and X-band EPR absorption spectroscopy after ascorbate treatments. (**a**) Progress curves for oxidation of 100 µM ascorbate in 20 mM MES pH 6.6 at 26 °C with 1 µM PfCopC (blue), PfCopC_E27A_ (black), PfCopC_H3F_ (blue dots), PfCopC_A2P_ (black dashes), Bim1 (green), TaAA9A (purple) loaded with 0.5 µM Cu(II). (**b**) EPR spectra of 500 µM PfCopC (blue), 250 µM PfCopC_E27A_ (black), 1.5 mM TaAA9A (purple) in the presence of 10 mM ascorbic acid.

The variants proteins PfCopC_A2P_, PfCopC_H3F_, and PfCopC_E27A_ were expressed similarly to the wild-type. Pro2 is found in PsCopC^26^ and Phe3 is found in MtCopC^27^. When tested in the ascorbate assay, PfCopC_H3F_ was indistinguishable from the wild-type protein (Fig. 7a) while PfCopC_A2P_ and PfCopC_E27A_ caused a constant rate of ascorbate oxidation. These slow but linearly progressive ascorbate oxidation profiles are different from both the profiles of PfCopC and TaAA9A. X-band EPR spectra were collected for PfCopC, PfCopC_E27A_ and TaAA9A with and without 10 mM ascorbic acid to ascertain if the proteins can accommodate Cu(II)-Cu(I) redox-cycles (Fig. 7b and SI Fig. 7a-c). As expected, the presence of excess ascorbate readily reduced TaAA9A to the Cu(I) state while the Cu(II)-PfCopC spectrum remained unchanged. Combined these data suggest that the ascorbate oxidation rate is limited by Cu(II) reduction rather than re-oxidation in Cu-PfCopC. Cu(II)-PfCopC_E27A_ behaves like the wild-type protein in these conditions and is not reduced by ascorbate within the timeframe of the experiment. However, the hyperfine Cu-nitrogen couplings that are clearly resolved in the Cu(II)-PfCopC are less defined in the spectrum of Cu(II)-PfCopC_E27A_ . This show that Cu-coordination is affected by the Glu residue in the second coordination sphere.

## Discussion

Three different representative members of Cu-histidine brace protein-classes were compared in this study. CopC (PfCopC), LPMO (TaAA9A) and the newly identified LPMO-like family X325 (Bim1) all contain the surface-exposed Cu-binding site formed by two imidazole side chains of histidine and the N-terminus.

### Differences in reactivity

PfCopC, Bim1 and TaAA9A are strikingly different in their reactivity. While TaAA9A produces oxidized cello-oligosaccharides from PASC, PfCopC and Bim1 do not. Arguably, cellulose is not a relevant substrate for periplasmic proteins such as PfCopC and Bim1, and both proteins lack any recognizable polysaccharide-binding site. Nonetheless, given suggestions by other over the years that LPMO action can be mediated by soluble activated species, it is important to demonstrate that CopC or Bim1 cannot produce species able to cleave a glycosidic bond in cellooligosaccharides, and this is tightly connected to the inability to initiate redox cycles.

### PfCopC is a strong Cu(II)-chelator

Cu chaperones such as CopC must prevent redox cycling before delivering Cu to the copper transporters in the plasma membrane^34^. Our data show that Cu(II)-PfCopC –in contrast to Cu(II)-TaAA9A- is not reduced by the biologically relevant reductant ascorbate. The result in the organism is safe transport of Cu(II) without generation of reactive oxygen species. In a recent crystallographic study of TpCopC, it was proposed that the polypeptide releases Cu(I) formed by photoreduction during exposure to X-ray. This conclusion was based on the proximity of one of the two CopC polypeptides (chain M) to a haem group of sulphide dehydrogenase with which it co-crystallised^28^. This observation suggests a mechanism in which CopC proteins bind Cu(II) very efficiently but readily releases Cu(I) if adequate conditions for copper reduction is met. In our study, this is likely the case in the condition with DTT. This conclusion is further supported by the inability of apo-PfCopC to compete with Bicinchoninic acid (BCA) for Cu(I) under anoxic conditions^23^. Furthermore, it is in agreement with findings from studies of PsCopC that has two copper binding sites each being highly specific for either Cu(I) or Cu(II), the Cu(II) binding-site being highly similar to PfCopC^26^. Our data show that apo-PfCopC can bind Cu(I) but with much lower affinity than the sub-femtomolar affinity for Cu(II) that has been reported^23^. Because apo-PfCopC has several possible Cu-configurations^23^, the Cu-coordination can in this case very well differ from the Cu(II)-histidine  brace.

It has been suggested that His3 is directly involved with Cu-coordination in PfCopC and a key to the very strong Cu(II) affinity^23^. The data presented here for PfCopC_H3F_ are incommensurate with His3 having such a role in Cu coordination in PfCopC. This is in agreement with recent findings^24,28^. Two other amino acid residues, namely Ala2^26^ and Glu27^28^, have been proposed to contribute to the strong binding of Cu to PfCopC. This was confirmed by the ascorbate assay, in which Cu-PfCopC_E27A_ and Cu-PfCopC_A2P_ both led to a slow oxidation of ascorbate, indicating that free copper is being released. Indeed the EPR spectrum show a less defined coordination of Cu to the nitrogens in Cu-PfCopC_E27A_. Further studies to probe the effect of individual amino acid side-chains on the Cu-chelating property of PfCopC amino acid are currently being carried out.

### Bim1 is distinct from LPMO and CopC

The findings presented here suggest that Cu(II)-Bim1 either oxidizes ascorbate at a very slow rate, or not at all. This is in agreement with the very limited reduction of Cu(II)-Bim1 during EXAFS analysis^5^. Also, the Cu-coordination geometry in the crystal structures of the structurally related protein LaX325 is consistent with Cu(II), and thus with no photoreduction of the metal by the X-rays during collection of the diffraction data^6^. The apparent acceleration of ascorbate oxidation by Cu(II)-Bim1 is instead caused by un-ligated copper being released from the histidine brace. The release of copper from Cu-Bim1 in this assay was induced by stoichiometric concentrations of H_2_O_2_. Native Bim1 is expressed under Cu-limited conditions as part of copper uptake mechanism^5^. The loss of Cu coordination upon reaction with H_2_O_2_ offers a mechanism for rapidly switching off copper import during oxidative stress. A similar mechanism has been shown to control the PerR transcription factor, which regulates the translation of peroxide defence genes^35^.

### TaAA9A and the free equatorial coordination position

TaAA9A has both a free equatorial position and a free axial position on the copper in the active site to bind reactants, the other axial position being occupied by a Tyr ligand as often seen in LPMOs. However, the free axial position may be blocked by a hydroxymethyl group of the polysaccharide substrate upon binding, as shown for *Lentinus similis* AA9A^36^. Tolman and co-workers have synthesized a series of square planar Cu(III)-complexes^37–39^ in which a common denominator is a free equatorial coordination site, and all the complexes are highly reactive. The equatorial position is thus in all likelihood the position where oxygen binds and reacts. All the equatorial positions are filled in both PfCopC and Bim1. It is relevant for this discussion to consider the putative active sites of *Methylococcus capsulatus* pMMO. Recently published work on pMMO has cast doubts on the nature of the active site of the enzyme^8^. The active site was first assigned to a di-nuclear copper site in subunit B, but later refined as a mononuclear site^40^. It has now been proposed that the active site is to be found in a different (C rather than B) subunit of the trimeric enzyme^41^. The new perspective of the B-site potentially being a redox inactive copper–histidine brace could possibly be explained by the third Cu-coordinating side chain blocking the equatorial position in this protein.

### Ascorbate oxidation by TaAA9A

LPMOs are reduced by a multitude of electron donors including ascorbate^4,42,43^ in the first step of catalysis and many reduced active site structures has been published^29^. Cu(I)-TaAA9A has been found to bind O_2_ and to form Cu(II)-TaAA9A-O_2_^-^ through an inner-sphere electron transfer^17^. The rate at which ascorbate is oxidized by Cu-TaAA9A is in good agreement with the reported Cu(I) re-oxidation rate (> 0.15 s^-1^). We note that the rate of H_2_O_2_ production in ascorbate solution by un-ligated copper is much higher than by Cu-TaAA9A. In contrast, the ascorbate oxidation rates are in the same range for un-ligated copper and Cu-TaAA9A. Furthermore, the very small effect of SOD and catalase on the rate of ascorbate oxidation by Cu-TaAA9A does not support quantitative displacement of H_2_O_2_ from the active site by water^44^. Neither does the small rate-increasing effect of SOD on H_2_O_2_ production by Cu-TaAA9A. This result support release of superoxide from the enzyme as suggested before^17^. However, a large proportion of the electrons from ascorbate are not accounted for in the produced H_2_O_2_. It is therefore conceivable that Cu-TaAA9A favours further reduction of the protein-bound oxygen species to water. The rate-increasing effect of BCA on ascorbate oxidation is interpreted as evidence of a highly reactive intermediate such as Cu(III) in the redox cycle as suggested by others^45^. Delocalisation and dynamic transfer of electrons between Cu and the TaAA9A polypeptide is likely^45,46^. However, these studies have been carried out in the absence of polysaccharide substrate and the importance of such electron transport and resulting amino acid radicals for cleavage of glucosidic bonds remains unresolved.

## Conclusions

Sequence based identification and classification of Cu-proteins such as LPMOs is at risk of being misleading. Small changes (on a biological scale) in the Cu-coordination sphere leads to dramatic changes in reactivity. This is a major challenge in elucidation of the in vivo function. The biochemical characterisation presented here is helpful in distinguishing between Cu-chaperones that function in Cu-import and enzymes. Furthermore, insight into the key structural factors that differentiate the proteins studied (PfCopC, Bim1 and TaAA9A) was provided: PfCopC is a strong Cu(II)-chelator due to partial deprotonation of the N-terminus by Glu27 and enabled by Ala2. Both residues are highly conserved. Bim1 binds Cu(II) less tightly, and copper thus leaks into the solution. This released copper catalyses classic Fenton chemistry, and is easily mistaken for enzyme activity. Bim1 is very sensitive to the resulting H_2_O_2_ leading to further release of copper to the solution. In contrast, Cu-TaAA9A can oxidize ascorbate directly without a free ROS intermediate. The mechanism by which TaAA9A oxidize ascorbate may involve a reaction intermediate that is quenched in a fast reaction with trace amounts of Cu(I).

## Methods

### Protein expression and purification

The synthetic gene of PfCopC (NCBI WP043048726) was optimized for *E. coli* (IDT) and subcloned into a pET28a-malE plasmid. The resulting pET28a-malEss-PfCopC was cloned in BL21(DE3) and grown in LB medium. Cells were grown at 30 °C with vigorous shaking, induced with 1 mM IPTG at mid-exponential phase and harvested by centrifugation after 20 h of expression. Proteins were extracted by mild sonication or isolation of the periplasmic fraction^47^. Site directed mutagenesis of pET28a-malEss-PfCopC was done using uracil excision cloning^48^. Cell extracts were passed through a 0.22 µm polyethersulfone filter and loaded on a Q Sepharose FF column (GE Healthcare) in 20 mM Tris buffer pH 8.3. PfCopC eluted in the flow-through which was collected and on loaded a SP Fast Flow column (GE Healthcare) after dilution to 40 mM MES buffer pH 5.6. Proteins were eluted with a linear gradient of 0–500 mM NaCl, and fractions containing PfCopC were pooled and concentrated on a 5 kDa centrifugal filter. PfCopC was further purified on a Superdex 75 gel-filtration column (GE Healthcare) in 40 mM MES with 150 mM NaCl. PfCopC purity at each step was tracked by SDS-PAGE and a 20% total recovery of the expressed PfCopC was estimated. The total expression yield was 27 mg of PfCopC per liter of expression medium.

TaAA9A was kindly provided by Novozymes A/S in the form of a frozen expression extract. The crude sample was thawed and passed through a 0.45 µm PES filter. The filtrate was diluted tenfold with 20 mM Tris buffer, pH 6.5, and loaded on a 60 mL Q Sepharose Fast Flow column (GE Healthcare). Proteins were eluted with a linear gradient of 0–500 mM NaCl and fractions containing pure TaAA9A were pooled and stored at 4 °C.

Bim1 was expressed with a C-terminal hexa-His tag in *Pichia pastoris* and purified as described elsewhere^5^*.* The tag was removed by cleavage with Tobacco Etch Virus protease (TEV) at a ratio of 1:40 Bim1 for 15 h at 4 °C. The TEV-protease and His-tag peptide were removed from solution using 100 µL Ni–NTA Agarose (Qiagen) equilibrated with 20 mM MOPS buffer, pH 6.72.

### Isothermal titration calorimetry experiments

ITC experiments were carried out in a 200 µL sample cell, 40 µL titration syringe 1.5–3 µL injection volumes and 150–240 s interval between injections using a MicroCal PEAQ-ITC (Malvern Pananalytical). The conditions used were 25 µM apo-proteins in 40 mM MES, 150 mM NaCl pH 6.6 at 25 °C. Data was fitted to a single-site binding model to determine condition dependent thermodynamic parameters and stoichiometry. For anaerobic experiments, the instrument was placed in a custom-made Belle Technology UK Ltd Rigid acrylic glovebox. Prior to use the glove box atmosphere was purged with N_2_ and maintained at < 0.8 ppm [O_2_] using a stand-alone recirculation unit using molecular sieves cartridges.

Preparation of anaerobic copper solutions were made similarly to^49^ but using CuCl as source of Cu(I): The solutions were made under anaerobic conditions in the presence of either MeCN, BCA or BCS. All buffers where extensively purged with N_2_ for 2 h while stirring in infusion flasks. Degassed buffers where kept in the anaerobic chamber for the duration of the experiments. Protein samples were first overlaid with argon gas for 2 min and then gas exchanged in the anaerobic chamber by stirring for 1 h at 300 rpm. MeCN was used to stabilize Cu(I) and prevent oxidation to Cu(II). Cu(II) content in copper solutions was measured by ITC titrations using EDTA as the titrants. Similarly, Cu(I) content was measured by using BCS as the titrant.

### Cellulose assay

Cellulose degradation assays were carried out in 400 μL samples containing 20 mM MES pH 6.6, 0.3% PASC, 1 mM ascorbic acid and 1 µM copper-loaded enzyme. The samples were prepared in 2 mL round bottom centrifugation tubes and incubated for 24 h in a thermomixer at 37 °C or 50 °C, with shaking at 650 rpm. Samples were filtered and analysed using HPAEC-PAD in the separation method described by Westereng et al.^50^.

### Absorbance and fluorescence spectroscopy

PfCopC was dialysed into 20 mM pH 6.6 phosphate buffer and prepared as 230–500 µM protein samples copper loaded with 0.8 equivalents of CuCl_2_. The samples were reduced with 1 mM ascorbate or 2.5 mM DTT at room temperature and loaded into 1 cm quartz cuvettes. Absorption spectra and tryptophan fluorescence spectra were measured every 5 min for at least 2 h on a Cary 50 (Varian) spectrometer. For anaerobic measurements, PfCopC was overlaid with argon for 2 min and transferred to an anaerobic glove box. The remaining oxygen was removed by gas exchange while stirring for 1.5 h. The sample was reduced with 1 mM ascorbate and loaded into 1 cm quartz cuvettes. The absorbance at 600 nm was measured every 5 min for 2 h on a FLAME spectrometer (Ocean Optics).

### Electron paramagnetic resonance spectroscopy

EPR spectra were measured on a Bruker EMX, 9.45 GHz on samples submerged in liquid nitrogen (77 K) using a microwave power of 10.0mW and modulation amplitude of 10Gauss. The samples were copper loaded to completion by overnight incubation in two-times excess CuCl2 and then buffer exchanged on 3 kDa Vivaspin 500 concentrators by 5 successive dilutions with 20 mM MES buffer pH 6.6 with 40 mM NaCl. The final protein concentrations were PfCopC 500 uM, PfCopC-E27A 250 uM, TaAA9A 1.5 mM. After acquisition, the samples where thawed and supplemented to 10 mM ascorbic acid and another set of spectra were acquired. Spectrum simulations were carried out in the MatLab plugin EasySpin^51^ using a spin system of 3 equal^14 ^N nuclei coupled to one copper.

### Ascorbate oxidation assay

Ascorbate oxidation assays were performed using a procedure similar to that described elsewhere^31^. Experiments were carried out in UV-transparent microtitre plates under ambient conditions. All solutions were prepared in 20 mM MES pH 6.6, and measurements were performed on a BioTek Synergy H1 plate reader. Copper binding was evaluated by equilibrating PfCopC, Bim1 and TaAA9A (2 μM) overnight with CuCl_2_ (0–2 μM). The assay was initiated by adding equal volumes of 200 μM freshly prepared ascorbate. The change in absorbance at 265 nm was followed in real-time for at least 1 h. Each condition was analysed at least in duplicate. Three separate experiments with TaAA9A gave a 1.2% standard deviation in the turnover frequency of ascorbate oxidation.

We tested the ascorbate assay with different oxygen species scavengers and deliberately copper-underloaded enzymes to ensure that no free copper was available. PfCopC, Bim1 and TaAA9A (4 μM) were equilibrated overnight with 2 μM CuCl_2_ and mixed with equal volumes of 8 μM BCA, 400 μM EDTA, 4 nM catalase, 40 nM superoxide dismutase (SOD) before the ascorbate assay was performed as described above.

We tested the ascorbate assay on the same plate reader in atmospheres of 1, 10 or 21% of molecular oxygen. A flow of nitrogen gas controlled by a retrofitted BioTek 121,008 unit maintained oxygen concentrations at the specified levels. The spectrometer chamber was preloaded with an assay plate and equilibrated for 3 h before injection of ascorbate using the built-in syringe channel and measurement as described above.

H_2_O_2_ assays were performed in the same way as the ascorbate assay, but with the Amplex UltraRed kit as an effector. The enzymes PfCopC, Bim1 and TaAA9A (4 μM) were equilibrated overnight with 2 μM CuCl_2_ and mixed with equal volume of 2 × Amplex UltraRed working solution. The change in fluorescence was followed in real-time with excitation at 530 nm and emission at 590 nm.

### Turnover frequency

Turnover frequencies were determined as the linear rate at the beginning of the progress curve v_0_ normalized by the concentration of enzyme and the spectroscopically signal amplitude. The full signal of oxidation of 100 µM ascorbate Δ was determined from the difference between copper-underloaded or overloaded PfCopC. The full signal of 100 µM of H_2_O_2_ was determined from a standard curve (see SI Fig. 4b)**.** The concentration of Cu(II) c was used to infer the concentration of holo-protein. The turnover frequency is reported as v = v_0_ /c * 100 µM/ Δabs.

## Supplementary information

Supplementary file1
