# Supplementary material for: Biochemical evidence of both copper chelation and oxygenase activity at the histidine brace
Source: Sci Rep. 2020 Oct 1;10:16369. doi: 10.1038/s41598-020-73266-y (PMC7529816; doi:10.1038/s41598-020-73266-y)
Supplement: Supplementary file 1 — Supplementary file1 [file 41598_2020_73266_MOESM1_ESM.docx]

Biochemical evidence of both copper chelation and oxygenase activity at the histidine brace

Søren Brander ^1^, Istvan Horvath ^2^, Johan Ø. Ipsen^3^, Ausra Peciulyte^4^, Lisbeth Olsson^4^, Cristina Hernández‑Rollán^5^, Morten H. H. Nørholm^5^, Susanne Mossin^6^, Leila Lo Leggio^7^, Corinna Probst^8^, Dennis J. Thiele^8^, Katja S. Johansen^1,4§^

^1^Department of Geoscience and Natural Resource Management, Copenhagen University, Frederiksberg, DK-1958, Denmark. ksj@ign.ku.dk

^2^Division of Chemical Biology, Department of Biology and Biological Engineering, Chalmers University of Technology, Kemivägen 10, SE-412 96 Gothenburg, Sweden

^3^Department of Plant and Environmental Sciences, Copenhagen University, Frederiksberg, DK-1871, Denmark

^4^Division of Industrial Biotechnology, Department of Biology and Biological Engineering, Chalmers University of Technology, Kemivägen 10, SE-412 96 Gothenburg, Sweden

^5^Novo Nordisk Foundation Center for Biosustainability, Technical University of Denmark, DK-2800 Kgs. Lyngby, Denmark

^6^Centre for Catalysis and Sustainable Chemistry, Department of Chemistry, Technical University of Denmark, DK-2800 Kgs. Lyngby, Denmark

^7^Department of Chemistry, University of Copenhagen, DK-2100 Copenhagen Ø, Denmark

^8^Department of Biochemistry, Pharmacology and Cancer Biology and Molecular Genetics and Microbiology, Duke University School of Medicine, Durham, 27710, North Carolina, USA

^§^Corresponding author

**Supplementary information**

Contents

[Isothermal titration calorimetry under anaerobic conditions, SI Figure 1 2](#_Toc48308801)

[Amino acid sequence alignment, SI Figure 2 3](#_Toc48308802)

[Cu-loading of TaAA9A, SI Figure 3 3](#_Toc48308803)

[H_2_O_2_ generation by free Copper and Cu-TaAA9A *Holo* complex SI Figure 4 4](#_Toc48308804)

[Absorbance and tryptophan fluorescence of PfCopC, SI Figure 5 5](#_Toc48308805)

[Conservation of a second sphere Glu, SI Figure 6 5](#_Toc48308806)

[EPR analysis, SI Figure 7 6](#_Toc48308807)

### Isothermal titration calorimetry under anaerobic conditions, SI Figure 1


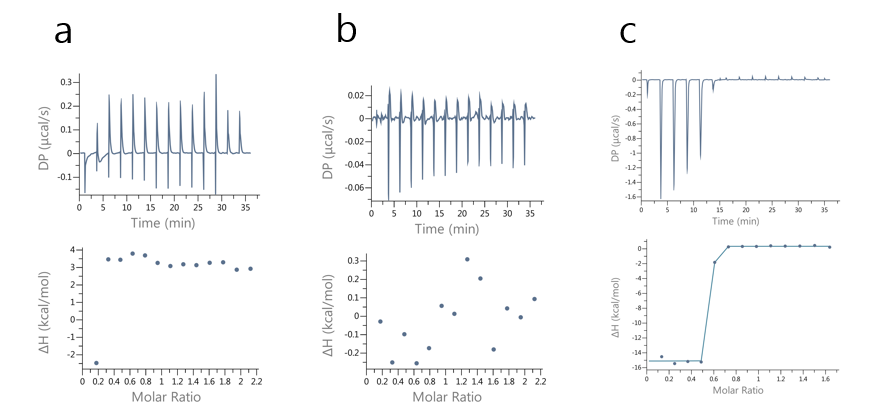


SI Figure 1: Anaerobic ITC titration of enzymes with Cu(I) in 40 mM MES, 150 mM NaCl pH 6.6 at 25°C. Cu(I) is insoluble in water and a titration experiment has to be carried out as a competition assay between two chelators. Titrations of apo-PfCopC and apo-TaAA9A with [CuI(MeCN4)]^+^ are given in the main text figure 3. Here we report the control ITC experiments a) Titration of 25uM PfCopC with the stable Cu(I)-(BCA)_2_ complex shows no competition. b) Titration of 25 µM [CuI(MeCN4)]^+^ with EDTA gives no signal and rules out the possibility of a Cu(II) contamination from disproportionation. c) Titration of 50 µM BCS with [CuI(MeCN4)]^+^ gives the expected strong titration at molar ratio of 2:1. The Chi-square fit parameters are:[CuI(MeCN4)]+ into TaAA9A: 6.0*× 10^-2^*, [CuI(MeCN4)]+ into PfCopC: 2.6*× 10^-2^*, and [CuI(MeCN4)]+ into BCS: 5.7 *× 10^-2^*. Due to differences in experimental conditions, the data are not directly comparable to the data from Johnson *et al* (D. K. Johnson *et al.*, Stabilization of Cu(I) for binding and calorimetric measurements in aqueous solution. *Dalton T* **44**, 16494-16505 (2015)). Graphs by the MicroCal PEAQ-ITC (Malvern Pananalytical) software.

### **
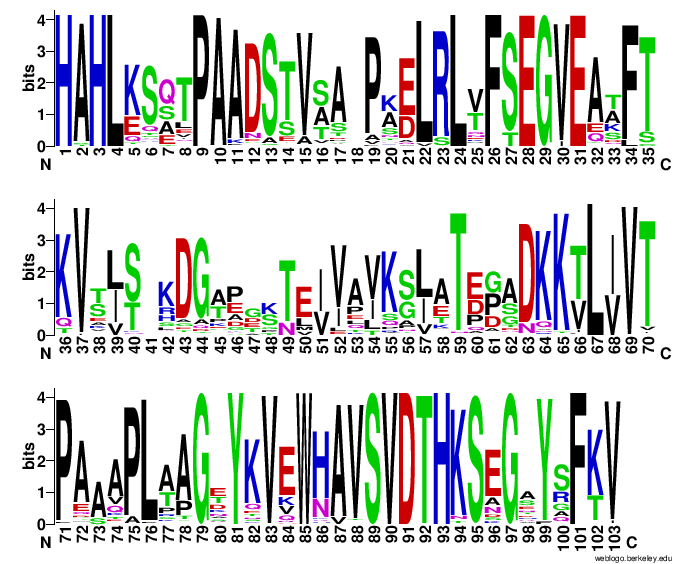

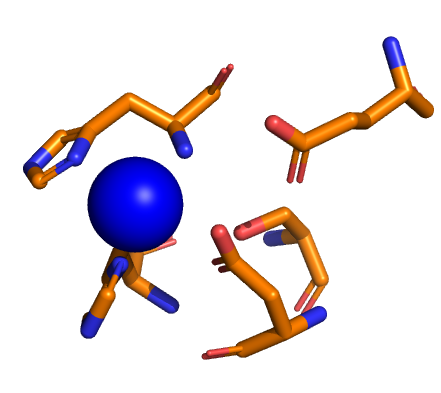
Amino acid sequence alignment, SI Figure 2**

D82

H85

E27

H1

SI Figure 2: PfCopC homologues were found by protein blast (<https://blast.ncbi.nlm.nih.gov/Blast.cgi>) using a 50% identity cut-off to the secreted amino acid sequence of PfCopC and UniRef90 as the sequence library. The resulting multiple alignment was visualised as a weblogo (<https://weblogo.berkeley.edu/logo.cgi> and the amino acids involved in copper binding is highlighted above (prepared in PyMol 2.3.1). In general, the conserved amino acids correspond to amino acids involved in core packing or binding of the copper metal. E27 in PfCopC (E28 in the logo alignment) makes only second sphere coordination to the copper, but the amino acids around this position are strictly conserved.

### Cu-loading of TaAA9A, SI Figure 3

b

a


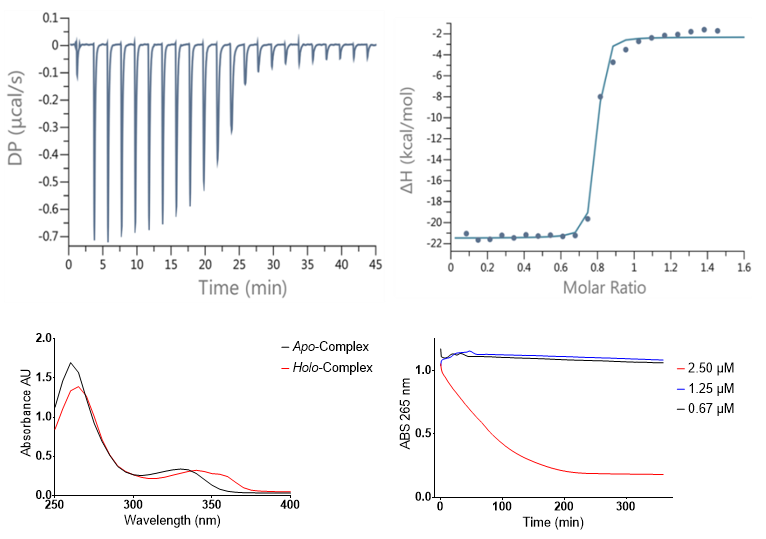


SI Figure 3: Determining Cu-content in TaAA9A. a) Thermograph of apo-TaAA9A titration with Cu (II) b) Data fitted to a single-site binding model with titration stoichiometry 0.75.

### H_2_O_2_ generation by free Copper and Cu-TaAA9A *Holo* complex SI Figure 4

b

a


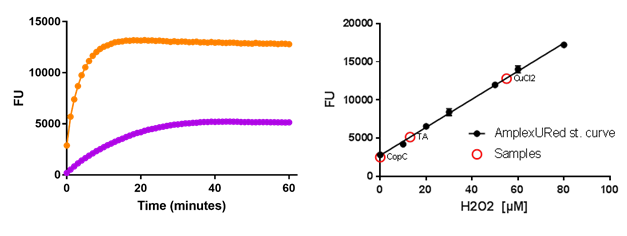


SI Figure 4: a) Progress curves of H_2_O_2_ production catalyzed by CuCl_2_ (orange) and Cu-TaAA9A (purple) in the Amplex Ultrared assay. b) Standard curve with the end point for CuCl_2_, Cu-TaAA9A, and Cu-CopC catalyzed oxidation of 100µM ascorbate indicated. The production of H_2_O_2_ from O_2_ requires two electrons and ascorbate contain two labile electrons per molecule. The efficiency of H_2_O_2_ production is thus approximately 58% of the theoretical by CuCl_2_ and 18% by Cu-TaAA9A.

### Absorbance and tryptophan fluorescence of PfCopC, SI Figure 5

SI Figure 5: Absorbance and tryptophan fluorescence of PfCopC and the effect of added copper, ascorbic acid and DTT. **(a)** The Cu(II) specific 600 nm band does not change after addition of 1mM ascorbate to 0.5mM aerobic (square) or 0.23mM anaerobic (triangles) Cu-PfCopC. This indicate that PfCopC is not reduced by ascorbate. **(b)** Absorbance spectra of 0.5 mM Apo-PfCopC (black) with added 0.8 equimolar CuCl_2_ (blue) and after addition of 2.5 mM DTT (red). The 600 nm band disappears with addition of DTT, which suggests a reduction of the copper (**c**) Tryptophan fluorescence spectra of same samples diluted 20 times. The fluorescence peak does not shift position, suggesting that the enzyme keeps the tertiary fold in all conditions. DTT removes Cu(II) quenching of tryptophan fluorescence which suggest a reduction of the copper. Ascorbate at higher concentration resulted in very low fluorescence due to inner filter effect. Ascorbate does not change the fluorescence peak of Cu(II)-PfCopC, showing that the Cu(II) is not reduced under these conditions.

**a**

**b**

**c**

### Conservation of a second sphere Glu, SI Figure 6

**e**


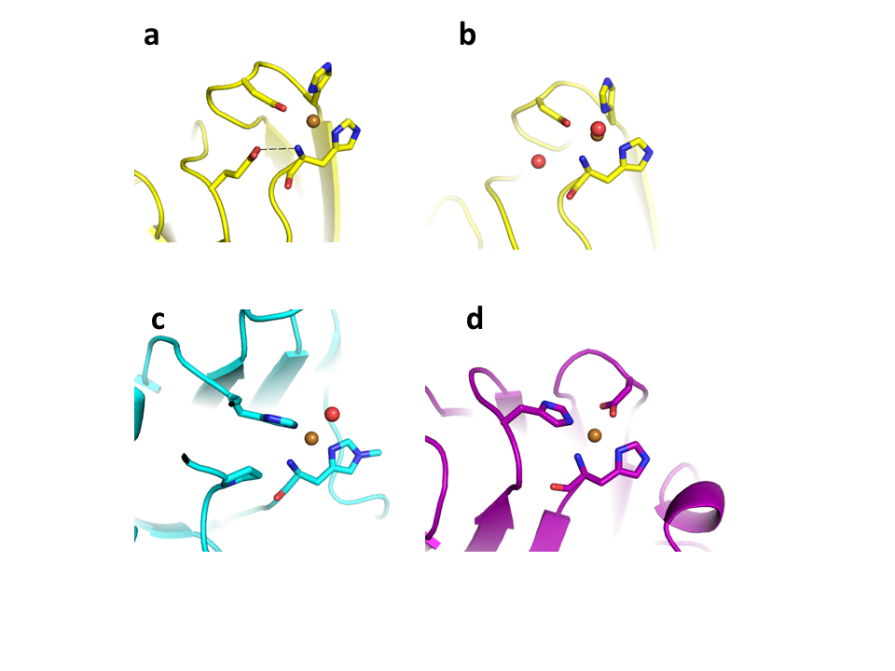

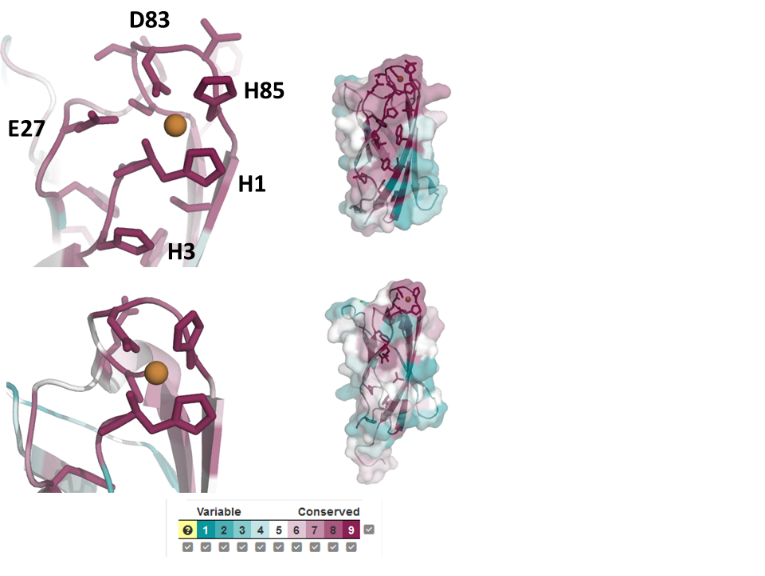


SI Figure 6: In CopC from *T. paradoxus* (PDB code 5N1T) the N-terminus interacts with a conserved Glu (a), but no corresponding acidic residues are found in CopC from *Methylosinus trichosporium* OB3b (PDB code 5ICU) (b), family AA9 LPMO A from *Thermoascus aurantiacus* (PDB code 2 YET) (c); X325 from *Laetisaria arvalis* (d). (e) Top: Consurf analysis of homologues (45%-95% sequence identical homologues from Uniprot-90 database) to CopC from *P . fluorescens* (PDB code 6NFQ). The His brace, Cu-coordinating Asp and N-term interacting Glu are very highly conserved (residues with 9 as conservation score are also shown as sticks – left is a close-up while right is an overall view with surface). Bottom: Similar analysis but with CopC from *Methylosinus trichosporium* OB3b (PDB code 5ICU). To ensure a sufficient number of sequences homologues with 40%-95% sequence identity were used. The equivalent of pfCopC Glu27 is a Gly in this structure and primarily Gly, Glu, Asn or Gln in the aligned sequences. Prepared in PyMol 2.3.1.

### EPR analysis, SI Figure 7

SI Figure 7: EPR spectra of 500uM PfCopC (a), 250uM PfCopC_E27A_ (b), 1500uM TaAA9a (c) before and after addition of 10mM ascorbic acid (+AA) as well as the simulated spectra of the untreated sample (sim). Addition of ascorbate does not change the spectra of PfCopC or PfCopC_E27A_ which clearly shows the CopC bound copper is not reduced by ascorbic acid. In contrast, TaAA9a is readily reduced by the ascorbate addition. PfCopC g∥=2.25, A∥=178G g⊥=2.06 and TaAA9 g∥=2.28, A∥=149G g⊥=2.06 of the oxidized samples are in agreement with published values. Superhyperfine copper-nitrogen couplings are resolved in the PfCopC spectrum and were best described from couplings with three similar nitrogens and A_N_∥=9.8G.The spectra of PfCopC_E27A_ fits the same parameters as PfCopC but the superhyperfine couplings are less defined. This difference shows that the copper coordination is more defined in PfCopC.
